# Features of Effective Medical Knowledge Resources to Support Point of Care Learning: A Focus Group Study

**DOI:** 10.1371/journal.pone.0080318

**Published:** 2013-11-25

**Authors:** David A. Cook, Kristi J. Sorensen, William Hersh, Richard A. Berger, John M. Wilkinson

**Affiliations:** 1 Office of Education Research, Mayo Medical School, Rochester, Minnesota, United States of America; 2 Division of General Internal Medicine, Mayo Clinic College of Medicine, Rochester, Minnesota, United States of America; 3 Knowledge Delivery Center, Mayo Clinic, Rochester, Minnesota, United States of America; 4 Department of Medical Informatics & Clinical Epidemiology, Oregon Health & Science University, Portland, Oregon, United States of America; 5 School of Continuous Professional Development, Mayo Clinic College of Medicine, Rochester, Minnesota, United States of America; 6 Department of Orthopedic Surgery, Mayo Clinic College of Medicine, Rochester, Minnesota, United States of America; 7 Department of Family Medicine, Mayo Clinic College of Medicine, Rochester, Minnesota, United States of America; University of South Australia, Australia

## Abstract

**Objective:**

Health care professionals access various information sources to quickly answer questions that arise in clinical practice. The features that favorably influence the selection and use of knowledge resources remain unclear. We sought to better understand how clinicians select among the various knowledge resources available to them, and from this to derive a model for an effective knowledge resource.

**Methods:**

We conducted 11 focus groups at an academic medical center and outlying community sites. We included a purposive sample of 50 primary care and subspecialist internal medicine and family medicine physicians. We transcribed focus group discussions and analyzed these using a constant comparative approach to inductively identify features that influence the selection of knowledge resources.

**Results:**

We identified nine features that influence users' selection of knowledge resources, namely efficiency (with sub-features of comprehensiveness, searchability, and brevity), integration with clinical workflow, credibility, user familiarity, capacity to identify a human expert, reflection of local care processes, optimization for the clinical question (e.g., diagnosis, treatment options, drug side effect), currency, and ability to support patient education. No single existing resource exemplifies all of these features.

**Conclusion:**

The influential features identified in this study will inform the development of knowledge resources, and could serve as a framework for future research in this field.

## Introduction

Physicians frequently identify gaps in their clinical knowledge, with estimates suggesting that clinical questions arise multiple times per day.[1], [2] Options to address such point of care (POC) questions include referral to a specialist, informal discussion with an expert, self-guided inquiry using knowledge resources, or not answering the question. Since the last option could result in inferior patient care, and the first two options increase costs, incur delays, or place demands on other providers and systems, physicians often initially attempt to answer the question themselves.

Yet self-guided inquiries are frequently unsuccessful.[2]–[4] Barriers to such inquiries include insufficient time, inadequate knowledge resources, excessive information, deficient search skills, and belief that an answer is not available.[2], [5]–[10] In recent years several electronic knowledge resources designed to facilitate physicians' POC learning have emerged (e.g., UpToDate, MD Consult, DynaMed, and PIER). Evidence suggests that such evidence synopses are at least equal to, and possible superior to, primary sources such as PubMed/MEDLINE,[11], [12] and that use may be associated with improved knowledge outcomes[13], [14] and patient outcomes including mortality and length of stay.[15], [16] However, these resources suffer from shortcomings in design[17], [18] and content.[18], [19] Physicians also use resources such as Google,[20], [21] personal notes, and informal peer contact (“curbside consultations”[22]). One meta-analysis identified integration into the workflow, providing specific recommendations, and evidence-based justifications as significant predictors of electronic knowledge resources' favorable impact.[23] However, those authors identified significant gaps in the literature and advocated further research targeting knowledge resource development and implementation.

Previous studies have used predefined quantitative questions (e.g. surveys) to evaluate physicians' knowledge resource preferences.[2], [24]–[26] However, both knowledge resources and the expectations of users evolve rapidly with changing technologies, such that past preferences may not be relevant to current or future applications. Research to understand the *reasons* underlying choices and usage patterns will contribute to a foundation upon which to design and implement knowledge resources. However, such studies are few. Two grounded theory studies identified several specific recommendations to improve knowledge resources, including comprehensiveness, efficient searching, and provision of explicit, actionable answers.[7], [27] Others have offered suggestions based on personal experience.[28] Given this limited evidence, further research into the optimal design and implementation of knowledge resources appears warranted.

## Objective

This report is part of a qualitative research study of physician learning in practice. Our first report focused on the barriers, enablers, and process of physician POC learning in general.[5] In the present study we extend those findings by concentrating on the knowledge resources that physicians use in POC learning. Specifically, we sought to better understand physicians' perceptions of the strengths and weaknesses of existing knowledge resources, and from this to derive a model for an effective knowledge resource. We define a knowledge resource broadly as a tool or artifact that facilitates the acquisition, assimilation, and application of information to facilitate medical decision-making, including synopses of primary literature and search tools that facilitate identification of other resources.

## Methods

We conducted a series of focus groups followed by grounded theory analysis.

### Ethics statement

The Mayo Clinic institutional review board deemed this study exempt from full review. We recruited participants via email. We obtained verbal consent at the start of each session as approved by the review board, and documented this in the session minutes (written consent is not required by our review board for studies of this type).

### Focus groups

From October 2011 to February 2012 we conducted 11 focus group sessions at an academic medical center and four affiliated primary care sites (see Table 1). At the start of each session the moderator read a brief clinical scenario describing a hypothetical patient with “several medical issues.” The moderator then noted, “You realize you’re uncertain about how to proceed with managing a specific issue,” and asked participants, using an interview guide,[5] to explain how they resolve such uncertainties.

**Table 1:** Session and participant demographics.

Session	Location*	No. participants	Men	IM†	FM†	Other specialty†
1	AMC	5	2	5	0	4
2	AMC	5	3	5	1	3
3	AMC	5	3	5	0	3
4	Community site 1	3	1	0	3	1
5	Community site 2	5	5	0	5	1
6	Community site 3	2	1	1	1	1
7	AMC	3	2	0	3	0
8	AMC	6	6	6	0	6
9	AMC	6	4	6	0	4
10	AMC	6	4	0	6	2
11	Community site 4	4	2	2	2	0
Total	-	50	33	30	21	25

* Location: AMC = academic medical center. Community sites were 30 to 70 miles from the AMC.

† IM = internal medicine; FM =  family medicine. Twenty-five participants were certified in one or more subspecialties, including adolescent medicine, allergy, cardiology, critical care, endocrinology, gastroenterology, geriatrics, hematology, hospital medicine, nephrology, pulmonology, and rheumatology.

Focus group participants included 50 practicing physicians in a large multi-site health system (see Table 1), selected through purposive sampling to reflect diverse backgrounds (internal medicine and family medicine physicians; generalists and subspecialists; and academic and community practitioners). We determined sample size using thematic saturation: after the first six focus groups, and after every one or two thereafter, we reviewed moderator notes and transcripts to identify newly-emergent themes. We stopped scheduling sessions when no new themes emerged.

### Context: accessible resources

All providers in our health system enjoy free access to a variety of commercial online knowledge resources including UpToDate (a physician-authored resource), MD Consult (a compilation of full-text journal articles, medical references, and drug information), and Micromedex (a collection of databases focused on drugs and toxicology). They also have access to a locally-developed evidence-based resource, AskMayoExpert, that contains care process models (algorithms describing ideal care pathways), answers to frequently-asked clinical questions, and contact information for topic experts.

### Data analysis

After each session participants completed a brief questionnaire about their use of online resources. Each session was audio recorded and transcribed verbatim except for masking of participant names. We analyzed the transcripts using the constant comparative method.[29] Investigators DAC, KJS, and RAB first inductively identified main themes in a high-level review of raw transcripts. Investigators DAC and KJS then coded all transcripts, iteratively refining (merging and splitting) the initial themes to accommodate emergent concepts, and continuously contrasting themes and excerpts to identify interrelationships among themes. We then consolidated these themes into a coherent model that parsimoniously reflected these interpretations. We used Dedoose (www.dedoose.com) to facilitate the coding and analysis.

## Results

### Participants' use of knowledge resources

In a brief survey at the end of the focus group, nearly all of the participants (46/50) reported using one or more online references at least twice weekly. These included UpToDate (N = 37), PubMed or MEDLINE (N = 18), specific online journals (N = 10), and AskMayoExpert (N = 10). Half (N = 25) reported using a mobile device (smartphone, tablet computer, etc) on a daily basis for work-related activities.

### Strengths and weaknesses of specific knowledge resources

Participants noted strengths and weaknesses for several specific knowledge resources, as summarized in Table 2 (see Table S1 for supporting quotations). Notably, the online resource UpToDate was by far the most commonly cited in both our survey (above) and in focus group discussions. While physicians lauded UpToDate's comprehensiveness, grounding in evidence, and relatively efficient navigation and search functionality, they generally agreed that UpToDate was far from perfect. Most notably, they perceived this resource generally presents too much information, and the information occasionally fails to answer common clinical questions.

**Table 2:** Strengths and weaknesses of specific knowledge resources.*

Resource	Strengths	Weaknesses
AskMayoExpert	• Reflects local practices• Is credible• Is quick, concise, accessible• Contains care process models, unifies the practice• Lists experts and contact information• Clarifies when to get a consult• Outlines first/next steps in getting a consult• Is improving in search function and content	• Has poor search and navigation functionality• Has insufficient breadth (topical coverage)• Has insufficient depth (within a topic); is not written for specialists• Was implemented incrementally (poor initial impression)• Uses a question-answer format• Doesn’t present evidence
UpToDate	• Finds answers quickly (efficient search, well-organized)• Has comprehensive breadth (topics)• Offers in-depth coverage (within a topic)• Includes a brief summary• Cites evidence; bibliography• Uses expert experience when evidence is incomplete• Is current	• Is often too long• Doesn’t describe local procedures (processes, test names)• Has some gaps in coverage (non-IM specialties)• Cannot answer complex questions
MD Consult	• Offers access to traditional textbooks (online)• Is accessible (on library website)• Contains useful patient education materials	• Has poor search and navigation functionality• Does not facilitate review of surrounding topics (“keyhole effect”)
Google	• Is quick• Is familiar to users• Identifies material (especially images) useful for patient education• Can identify information using related terms	• Identifies material of variable credibility
PubMed, MEDLINE, Google Scholar	• Facilitates answering focused questions, rare conditions, • obscure topics• Can find a specific article or guideline• Offers information of known currency and credibility• Enables access to full- or partial-text publications• Is familiar to users	• Is time-consuming• [Google Scholar] brings up older articles
Printed materials (textbooks, article reprints)	• Are familiar and consistent; visual memory helps with search and retention• Are respected and credible• Facilitate review of surrounding topics, i.e., no keyhole effect (see MD Consult above)• Are not dependent on new technologies	• Are often less accessible
Personal notes	• Are familiar, personalized, and relevant• Are quick• Can be implemented using various technologies	• Are difficult to keep organized, find information• May be less accessible
Specific Internet sites	• Offer information useful to patients; empowers patient to answer their own questions (from specific sites)• Offer patient handouts (from specific sites)	
Micromedex	• Is focused on specific type of information (pharmacotherapy)	
Mobile devices	• Are always available	• Suffer from small screen• Require different applications with change in mobile operating system platform
Electronic medical record	• Is integrated into workflow: efficient, relevant	• Is sparsely implemented thus far

* Table S1 contains quotations to support the above strengths and weaknesses.

The locally-developed AskMayoExpert resource (see description in Methods) was discussed by many physicians as the chief competitor to UpToDate for answering clinical questions at the POC. Concise, practical answers and relevance to local practices were noted as the chief strengths, but spotty topical coverage and lack of explicit reference to supporting evidence/literature were cited as weaknesses. Physicians also noted difficulty navigating or searching the site. Several physicians disliked the question-and-answer format, although others noted this as a strength.

Google was often cited as the next source. Physicians appreciated its speed, availability, intuitive search, and access to images and other resources. However, they expressed concern about the variable credibility of the sources. MD Consult was also frequently noted, but typically as a third- or fourth-line resource because of its difficult navigation and search functionality and because of the “keyhole effect,” i.e., presenting a narrow slice of information such that piecing together a complete answer is difficult. Online literature indices such as PubMed and MEDLINE were frequently mentioned, but because of the time required to search for and digest information they were rarely described as useful in POC learning, unless the physician knew a priori of a specific article (e.g., a clinical guideline). However, for learning after the patient left or for general learning needs, physicians noted that literature indices possess numerous strengths including credibility, currency, and capacity for obscure questions. Several physicians noted Google Scholar as a quick way to find credible information, including full-text journal articles and guidelines.

Despite the growing presence of electronic resources, several physicians noted continued regular use of print materials such as textbooks and article reprints. One of the most consistent strengths of these resources is physicians' familiarity with these texts, which in turn facilitates rapid identification of needed information and (for information they had previously studied) learning reinforcement. Participants also described the benefit of stumbling across information on a nearby page that informs their decisions, but that they might miss in a targeted (i.e., electronic) search (the opposite of the keyhole effect). Challenges in using print materials as point-of-care resources include their physical inaccessibility (generally felt to be a minor concern) and the volume of information in most textbooks.

Finally, many physicians use personally-developed resources on a regular basis, both print (e.g. index cards) and electronic (on a mobile device, e-mail folder, or computer server). Advantages of personal resources include high familiarity and speed of access, relevance to needs, and a personalized organization structure.

### Features of effective knowledge resources

Drawing on the strengths and weaknesses noted above, together with other comments, we identified nine key features of useful knowledge resources (see Table 3).

**Table 3:** Key features of effective knowledge resources.

• Efficient: comprehensive, searchable, and brief• Integrated with clinical workflow• Credible, evidence-based, and practical• Familiar to user• Capable of identifying local human expert• Reflective of local care processes• Optimized for needed role• Current• Supportive of patient education

#### Efficient: Is the answer there? How quickly can I find it? Is the answer succinct?

First and foremost, and in keeping with the chief barrier of insufficient time, physicians desire efficiency. To this end, many physicians explicitly identified the *likelihood of finding their answer* (i.e., the completeness of topic availability and relevance of content) as the driving force behind their selection of resources:

When you have a choice of multiple resources to go to, what you want to know is kind of very quickly, 1) is the answer here? And you want to spend as little energy possible finding out is the answer here or not; and … 2) how quickly can I get to it through reliable search? (Session 8)

The reason I used UpToDate is because it’s reliable. I know that typically it will be well populated, the information will be there and if the information is not there, then it’s unlikely to be in other places. (Session 8)

Another aspect of efficiency regards the *speed with which the answer can be found* within a given resource. This in turn involves at least three considerations: 1) the organization and search functionality, 2) the length of content, and 3) familiarity with the resource (discussed in detail below). Poor search and navigation functionalities were often cited as reasons not to use a given resource. Excessively long content was likewise cited as a barrier, although when forced to choose nearly everyone would opt for longer (and more complete) content rather than overly brief (such that the question remains unanswered). Familiarity with a resource enhances both finding and reading the answer.

UpToDate – the search engine – is more efficient, spot on. You can type something and you can find it; it’ll be at the top of the list. (Session 2)

The thing I like about AME is that it’s very concise and very small. But it’s got a lot of links in there, too, so you can drill down if you want to. … It’s faster because UpToDate is really quite expansive. (Session 3)

One of the problems with too many sources is if you’re not used to using them, then it takes more time than it’s worth to try to find out how use them in the middle of the game. (Session 8)

While not explicitly reflected in any single quote, we noted a clear tension between these dimensions of efficiency: physicians *desire* to find the relevant topic quickly but they *require* comprehensive topical coverage; they *want* to avoid reading a lot of text, but they *need* detailed information. Refined search functions are essential. The use of layered information, for example an initial succinct summary with optional in-depth, evidence-supported detail, was viewed favorably (see quote above).

Even though it’s expanded a lot in the last year, I still don’t think it has the scope of … UpToDate. That’s why I don’t go there first: because … unless I know it’s got a section in there, I don’t want to spend the time having to query it before I then go to UpToDate. (Session 3)

Sometimes the information that’s present is just too basic. It’s not deep enough and so you’re needing to go to a second or a third source. … You can use it effectively often enough that you go back to it, but it likely won’t satisfy your needs every time. Whereas … the UpToDate system - a little bit more robust with the bibliography - more often it will meet your needs the first time. It’s an easier search function. (Session 10)

Despite the ubiquity of electronic resources, physicians recognized that these are not a panacea for time pressures. Personal notes (both printed and electronic) and print textbooks still play a significant role in quickly answering clinical questions. Moreover, as one physician noted:

It’s neat that we have the resources a little more accessible than our “ancient” text books, but we still have to figure *when* to review it. (Session 7)

#### Integrated with clinical workflow

Physicians recognized that one solution to the problem of finding the information would be for information to come to them when needed – built into their clinical workflow – through better integration of knowledge resources and the electronic health record.

You get these funny fungal cultures back from bronchoscopy … and the lab report reads out *Candida “blah”*, and I think, “What is that?” If there was just a button that said, “Candida blah – AskMayoExpert,” and you could get one click away from a paragraph, “What is this bug,” I’d click it every time, and that’d be the first thing I’d go to. (Session 8)

If we’re gonna tie the practice together, then we gotta find a way to make sure that it’s right in front of us and easy to access. (Session 5)

#### Credible, evidence-based, and practical

Physicians seek a credible knowledge source – but mentioned this requirement far less often than the need for efficiency. It appears that physicians satisfy the credibility of the resource early on, during the initial selection of a resource, and thereafter implicitly trust the resources they recurrently use. Indeed, when physicians did mention credibility it was most often in relation to a new, untried resource (as might be found in an Internet search). They identified four approaches to determining the credibility of an unfamiliar resource: 1) agreement of information with the physician's prior knowledge; 2) triangulation (finding the same answer in a second resource); 3) reference to the literature or actual evidence (study data) presented in the narrative; and 4) credible sponsor (university, government, or pharmaceutical source).

You typically have about a 90 or 85% confidence interval. OK, you’re pretty sure of what you’re going to do. … It’s not like you’re fumbling around in the dark, trying to find some piece of information. You know what to expect in the answers. (Session 10)

You look through 3 or 4 or 5 sources and if 4 of them say the same thing, then you can tell who’s an outlier. (Session 10)

I’m not going to take that paragraph at face value but I’ve got to click the link to the PDF right there and I can go look up the source literature if I want to, and I can figure out if the UpToDate author has kind of characterized that in an accurate way. (Session 3)

I will actually say in defense of drug companies – for professional sites – they’re … reviewed by the FDA and so they are like the same as the package insert. (Session 10)

Although evidence-based answers are highly prized, physicians also value practical answers even if the resource author must extrapolate beyond current evidence (provided such extrapolations are explicit).

I looked up a case of complicated Bell's Palsy and … [the evidence on antivirals] showed that you shouldn’t in most people, but in the advanced cases there wasn’t enough to prove or disprove, and so they said it may be worth considering it. So they used wording that sort of protected themselves, but they went out on a limb enough to kind of tell you what to do in the next level of more ambiguous or clinical concern. (Session 10)

#### Familiar to user

Physicians typically have a favorite resource for a given question type – often dating back to residency training – and are reluctant to adopt or even try unfamiliar resources. Familiarity seems to relate to their expectation of success (have I found answers to similar questions previously?) and their comfort navigating the resource (organization and search function). Many physicians still rely on self-authored resources (e.g., personal notes) or on older print resources because they can locate answers therein more quickly than with newer resources.

I’m just used to other things, like PubMed. It’s not that it’s better than AskMayoExpert, just what I’m used to and we’re creatures of habit. (Session 1)

UpToDate was the best source when I was a resident training and at two in the morning when you wanted a quick answer to get things going, that was the resource. … So I got trained into UpToDate, I guess. (Session 3)

Some participants felt that lack of familiarity impacts not only speed, but also learning effectiveness.

I’m not accessing the same information that I used to, … and so I don’t think I’m holding onto it as well. For instance, … as an intern I would refer to my MGH pocket guide. … I knew exactly where to flip to; I didn’t need the index and I knew exactly where on the page to look for that information, and it was sort of just on the tip of my tongue, or my mind. … But now, when I’m looking at different resources, it doesn’t come back as quickly, that stored memory. … [The pocket guide] was a consistent source that didn’t change. ‘Cause now if I go to UpToDate, the article has been changed; or if I go to the literature there is a more recent article. … It’s updating me, but it’s not as hardwired in my mind. (Session 9)

#### Capable of identifying an expert: Can I talk with a human?

For very complex questions, physicians often seek to contact an expert and appreciate the names and contact information for local experts available in AskMayoExpert (see also Table 2).

It’s not just the one question; it’s in the context of other things that the book doesn’t know. And maybe the answer there is right for an isolated situation. The problem is all of our patients are complex. …. So you’re trying to put this in the context of other things where it interacts with other areas; and that’s where the interaction [of talking with an expert] … is more helpful. (Session 8)

#### Reflective of local processes

Physicians encounter occasional difficulties translating the advice in commercial knowledge resources into concrete next steps at a local level due to variation in local care processes, alternate names for specific medical tests, or practical questions such as “What tests should I order before requesting a formal consultation?”

Sometimes trying to figure out what I need to order *here* (versus what the current UpToDate is telling me to order or do) can be a little different. (Session 9)

#### Optimized for needed role

Physicians often preferred different knowledge resources depending on the specific question. For example, while UpToDate was by far the most cited resource overall, most physicians suggested another resource such as Micromedex or Epocrates when searching for information on drug dosing or side effects. Each resource also has specialty-specific strengths and weaknesses. Algorithms and flow diagrams were noted as useful in describing care processes.

It might be a very simple thing about dosing or something in which case you might go to Micromedex and just look at the doses and drug interactions and side effects. (Session 10)

UpToDate [is] not as great in terms of pediatrics and ObGyn, not as great in orthopedics. (Sesssion 4)

#### Current: Is it up-to-date?

Physicians rarely mentioned the currency of information. We do not know whether physicians presume currency in modern knowledge resources, consider it less important, or simply discussed other topics.

Current, so, you know, it wouldn’t help me much to have a page that’s 2 years old. So it’s got to be something that’s regularly updated and fresh. (Session 8)

#### Supportive of patient education

Finally, physicians often use knowledge resources for patient education. They frequently use Google for images, UpToDate when discussing evidence-based treatments, and MD Consult or mayoclinic.com for patient handouts.

I tell them, “I haven’t seen this for a while, let’s look it up and read about it,” and then I pop it up and we look at it together and talk about the latest treatment or latest recommendation. (Session 6)

I use Google images all the time, for rashes or an as an illustration. Cardiac problems? Pull an image to use to try to explain things. (Session 7)

I also sometimes use mayoclinic.com for patient education. … And showing the patient how to access that information so that they can be empowered to [know] where they can go for medical questions. (Session 7)

## Discussion

In this focus group study we identified the strengths and weaknesses of specific knowledge resources to support physician POC information seeking (Table 2), and from this developed a list of features (Table 3) that appear to define an effective resource. We defined knowledge resource very broadly in this analysis, but we expect that these key features will apply most readily to purpose-built electronic resources. While several existing resources incorporate many of these key features, none embody all of these features.

### Limitations and strengths

These features do not define an *ideal* knowledge resource, but do propose criteria for *more effective* resources. Our study design did not permit objective evaluation of the relative merits of each feature; this could be the subject of future research. While the specific comments referring to a proprietary system (AskMayoExpert) are of limited generalizability, they provided a useful contrast with other resources and thus informed our model; we have already made substantial improvements to this system in response to these comments. Physician opinions might not necessarily indicate effective practices, and we did not triangulate our focus group data with other observations. Strengths of this study include the rigorous application of qualitative techniques, and the purposeful involvement of primary care and specialist physicians in both academic and community settings.

### Integration with prior work

Studies have evaluated knowledge synthesis resources in comparison with other knowledge resources,[30]–[33] PubMed,[11], [12], [33] and no specific intervention.[13], [15], [16] Others have evaluated the topical coverage, evidence base, and currency of various resources.[18], [19], [34]–[37] However, none of these studies systematically evaluated or identified important design characteristics.

Ely[7] identified 22 recommendations to improve knowledge resources, clustered in the categories of comprehensiveness, trust, navigation, clinical organization, and accessibility. Our work complements this prior work by re-emphasizing several common recommendations and by adding new key features: integrated, familiar, capable of identifying the human expert, and reflective of local care processes.

Given the prevalent use of Internet resources like Google,[20], [21] it seems appropriate to study how to judge the quality of the information thus obtained[38] and how to train physicians to make such judgments.[39]

Clinicians have difficulty answering complex questions using electronic knowledge resources.[27], [37] While one solution is to simplify the question,[27] participants in our study indicated that for complex questions they typically contact a human expert. Expanding the definition of knowledge resources to include curbside consultations[22] enables new opportunities to facilitate knowledge transfer and POC learning.

Previous studies have identified several barriers to physician information seeking other than the availability and functionality of knowledge resources, including insufficient time, patient complexity, inadequate search skills, the sheer volume of information available, and belief that an answer is not available.[2], [5]–[10] Providing access to robust knowledge resources without addressing these other concerns may be insufficient to improve POC learning.

### Conclusions and implications

Given the growing volume of medical information,[40] physicians will of necessity increasingly turn to evidence syntheses[41] to guide practice change. Although the rigorous grading and distillation of such evidence is essential, the manner in which this information is then communicated to physicians is likewise important.[27] The features identified in this study offer guidance regarding how this might be effectively done. While our model does not rise to the level of theory, it nonetheless incorporates and integrates several disparate perspectives of informatics and clinical decision-making including decision support, human factors, evidence-based practice, local care variation, point-of-care learning, and inter-physician communication. Concurrent with design improvements, developers might evaluate the comparative effectiveness of the proposed features to confirm their merit and determine their relative costs and benefits.

## Supporting Information

Table S1 Strengths and weaknesses of specific knowledge resources. This table contains quotations to support the strengths and weaknesses noted in the corresponding section of the Results.
(DOC)
