# Supplementary material for: Features of Effective Medical Knowledge Resources to Support Point of Care Learning: A Focus Group Study
Source: PLoS One. 2013 Nov 25;8(11):e80318. doi: 10.1371/journal.pone.0080318 (PMC3840020; doi:10.1371/journal.pone.0080318)
Supplement: Table S1 — Strengths and weaknesses of specific knowledge resources. This table contains quotations to support the strengths and weaknesses noted in the corresponding section of the Results. (DOC) [file pone.0080318.s001.doc]

**Features of effective medical knowledge resources to support point of care learning: A focus group study**

**Appendix Table S1. Strengths and weaknesses of specific knowledge resources**

| **Resource** | **Attributes** | **Quotes** |
| --- | --- | --- |
| AskMayoExpert | **Strengths**   - Reflects local practices - Is credible - Is quick, concise, accessible - Contains care process models, unifies the practice - Lists experts and contact information - Clarifies when to get a consult - Outlines first/next steps in getting a consult - Is improving in search function and content   **Weaknesses**   - Has poor search and navigation functionality - Has insufficient breadth (topical coverage) - Has insufficient depth (within a topic); is not written for specialists - Was implemented incrementally (poor initial impression) - Uses a question-answer format - Doesn’t present evidence | **Strengths**  One of the things that great about AME is that … there are certain Mayo ways of doing things and that’s laid out in there. (Session 10)  By being developed by Mayo we assume that there’s a level of credibility that goes along with whatever answers are in there, and I think that’s probably a fair assumption. (Session 8)  There’s so limited time. That’s where I find AME very helpful, because oftentimes I can get to that answer pretty quickly. … I don’t want it to duplicate what’s already out there. I like it to have its unique style. I wouldn’t want it to be another UpToDate, for instance. (Session 3)  The thing I like about AME, is that it’s very, very concise and very small. But it’s got a lot of links in there, too, so you can drill down if you want to. … Sometimes those frequently asked questions are the ones I’m asking. … UpToDate is way easier to get through then a chapter in Harrison’s; however, AME is even quicker. … And then it also allows you to go to that colleague. (Session 3)  I really like these Care Process Models … Some of them are very useful and maybe it’s because of the way I organize information in my head, but it’s nice to see flow diagrams where applicable. I think AME has done a very nice job with some of those offerings. Perhaps better than UpToDate. (Session 8)  If we’re gonna tie the practice together, then we gotta find a way to make sure that it’s right in front of us and easy to access. (Session 5)  I do use MayoExpert … to find out who in this specialty has an interest in this area. That’s the person I am gonna email or call. I think it is very helpful for that. (Session 2)  Some of the most helpful things that I’ve found in there, again as a generalist, [are] the statements that say, … “If it’s anything beyond this, they really should be seen by Cardiology.” (Session 2)  How to get in referrals - “Here’s some qualifications; here’s some things you can do; this needs to go first, this one can wait.” - type of thing. I think that’s got some potential. [Other participant: How to navigate the Mayo environment.] Right. “Here are the three [key] symptoms.” [or] “If you have this, you probably need these set of labs.” (Session 2)  This is more and more happening to me, where the very top question was the one that I was interested in. (Session 3)  **Weaknesses**  When I type in a keyword like I would for UpToDate, it’s just not as easy to navigate. (Session 9)  I think that the search function and the way you find the information is just cumbersome in AskMayoExpert. That’s probably my barrier to using it. (Session 10)  Even though it’s expanded a lot in the last year, I still don’t think it has the scope of what something like UpToDate has. … I don’t want to spend the time having to query it before I then go to UpToDate. (Session 3)  I just found the topics were limited. So there was a Derm thing I wanted, but they don’t normally have Derm on there, so I’m like, “OK, well I can’t use that.” So, … then I stopped going back and I just go to UpToDate. (Session 11)  Why have I adopted the habit of using UpToDate rather than AskMayoExpert? Probably because AskMayoExpert was populated incrementally as it rolled out and so you kind of had to know, “Is it going to be there? Has that section been discussed or not yet?” And so I got into a habit of just going to UpToDate, and haven’t broken that. (Session 8)  Sometimes the information that’s present is just too basic. It’s not deep enough and so you’re needing to go to a second or a third source, and so that is one of the dilemmas. And you can use it effectively often enough that you go back to it, but it likely won’t satisfy your needs every time. (Session 10)  It’s written not really for subspecialists, it’s written for NPs and PAs and maybe internists. (Session 8)  What I’ve sensed from AME is that in the effort to be concise they tend to skimp on the quantitative answers to the question. And so they give a description of “here’s the plan,” “here’s what you use,” but then the background stuff … is kind of left out. (Session 3)  I don’t feel like often I have time to dig through all of the questions to find the answer that I wouldn’t want to look through. For example, it’s the question format in AME and so sometimes that’s a bit burdensome. When in UpToDate, it’s more easily, sometimes it’s more easily retrievable. (Session 8) |
| UpToDate | **Strengths**   - Finds answers quickly (efficient search, well-organized) - Has comprehensive breadth (topics) - Offers in-depth coverage (within a topic) - Includes a brief summary - Cites evidence; bibliography - Uses expert experience when evidence is incomplete - Is current   **Weaknesses**   - Is often too long - Doesn’t describe local procedures (processes, test names) - Has some gaps in coverage (non-IM specialties) - Cannot answer complex questions | **Strengths**  UpToDate I think is the quickest way to get what I’m looking for in a pinch. (Session 2)  UpToDate is - the search engine is - more efficient, spot on. You can type something and you can find it; it’ll be at the top of the list. (Session 2)  The reason I used UpToDate is because it’s reliable. I know that typically it will be well populated, the information will be there, and if the information is not there then it’s unlikely to be in AskMayoExpert or other places. (Session 8)  UpToDate is probably a little bit more in-depth information. And so if you have a more in-depth question or if you have more time, and you’re at a desktop, it’s probably easier to just do that. (Session 6)  You can scroll down to the bottom and it has highlights - 5 bullets for recommendations or summary of a 7-page article. (Session 1)  UpToDate is an outstanding resource and … it has a bibliography that really can direct you for deeper looks if you need. (Session 10)  UpToDate will give you that paragraph. … I’m not going to take that paragraph at face value but I’ve got to click the link to the PDF right there and I can go look up the source literature if I want to, and I can figure out if the UpToDate author has kind of characterized that in an accurate way. (Session 3)  [UpToDate authors] also go out on a limb more to offer opinions. So I looked up a case of complicated Bell’s Palsy and ... [the evidence on antivirals] showed that you shouldn’t in most people, but in the advanced cases there wasn’t enough to prove or disprove, and so they said it may be worth considering it. So they used wording that sort of protected themselves, but they went out on a limb enough to kind of tell you what to do in the next level of more ambiguous or clinical concern. (Session 10)  UpToDate I think generally is up to date. (Session 7)  It’s really hard to beat somebody who does it 24/7. (Session 2)  **Weaknesses**  I don’t necessarily love UpToDate. It's OK, but it’s got a lot you’ve got to go through to find the clinically relevant information that you want. … When I’ve got like very specific questions, I want very specific answers and I don’t want to have to read a bunch of stuff. (Session 4)  In some instances if you’re looking something in UpToDate the premise is a little different in terms of how tests are organized, and sometimes trying to figure out what I need to order *here* [at Mayo] (versus what the current UpToDate is telling me to order or do) can be a little different. (Session 9)  I’ve had that problem with UpToDate too. They’re not specific enough. (Session 11)  UpToDate not as great in terms of pediatrics and ObGyn, not as great in orthopedics and some of the other subspecialty areas. (Sesssion 4)  Having things like UpToDate … sometimes there’s still information or experience … that can’t be bypassed. And having an expert who’s seen this 20 times lay their hands or lay their eyes on someone; sometimes it’s a lot more comforting. (Session 2) |
| MD Consult | **Strengths**   - Offers access to traditional textbooks (online) - Is accessible (on library website) - Contains useful patient education materials   **Weaknesses**   - Has poor search and navigation functionality - Does not facilitate review of surrounding topics (“keyhole effect”) | **Strengths**  MD Consult’s great, so if you have even not much time, you can usually get your answer off of it really pretty quickly. …. I just kind of gotten used to using that myself. (Session 5)  MD Consult is a conglomeration of online textbooks, some of which I don’t have in my office, … and other more specific journal information that you can go ahead and access on a pretty rapid basis as well. So, I find that to be a very useful site. (Session 4)  MD Consult is what I use for patient ed[ucation] materials most I guess.(Session 4)  **Weaknesses**  Rarely [use] MD Consult. I don’t like it. I just don’t find it as easy to get around. (Session 6)  The electronic versions of these textbooks, Braunwald’s and others, are online, but they are not as helpful. When I pulled Braunwald’s off, and I looked up pericardial effusion, I would find out in the chapter before, in the few pages before, the diagrams. When I’d go to Braunwald’s [online] I got the keyhole effect. By looking at 1 page, I’m drilling into 1 page. I can’t sort, easily flip; the pictures are not as easily [accessible]. (Session 9)  [On MD Consult] sometimes you have to can your search words in a bit of an odd fashion and such, and try a couple of times before you really get to drill down to what you need. (Session 4) |
| Google | **Strengths**   - Is quick - Is familiar to users - Identifies material (especially images) useful for patient education - Can identify information using related terms   **Weaknesses**   - Identifies material of variable credibility | **Strengths**  Google has made it so easy to find whatever you need that it’s just easier to do it. (Session 9)  I use main Google sometimes. To get hits on papers and so on, it’s actually very quick and effective. Then I go back to MEDLINE so I’ll get that paper if I want full text. Google is very effective. (Session 8)  Even random Google searches, if you use medical specific-terms, you can often find guidelines and algorithms and things (Session 10)  I used [Google] a few times this morning. Chalazion, just before I came here. Person with meralgia paresthetica illustrated. So, it’s a necessary tool. (Session 7)  That’s why Google is, to me, so awesome because if I want to show a patient a picture of something, I can get to it so much faster by just Googling. (Session 4)  Googling a question can kind of take you to things I didn’t know that you could. (Session 4)  **Weaknesses**  [Person 1] Don’t you get a lot of garbage out there on Google?  [Person 2] Well, it depends on what you type in.  [Person 3] If you use Google Scholar you’ll get a lot less. (Session 2)  I may be entering a Google search and who knows where that is gonna take you. (Session 3) |
| PubMed, MEDLINE, Google Scholar | **Strengths**   - Facilitates answering focused questions, rare conditions, obscure topics - Can find a specific article or guideline - Offers information of known currency and credibility - Enables access to full- or partial-text publications - Is familiar to users   **Weaknesses**   - Is time-consuming - Google Scholar brings up older articles | **Strengths**  [With] PubMed you can ask something specific: “Find the most updated article that’s from a reputable source” (Session 1)  Those [complex cases] are the kind of patients we often see here. …There’s nothing in UpToDate about their issue; and [to] look then for the case reports or series you have to go to PubMed. (Session 1)  I must be the only person who likes to use these only to find the original articles … to solve my questions more than I trust the writer of UpToDate. (Session 3)  I’m just used to other things, like PubMed. It’s not that it’s better than AskMayoExpert, just what I’m used to and we’re creatures of habit. (Session 1)  Google Scholar is faster [than PubMed]. (Session 1)  If you use Google Scholar they’ll bring up tons of actual publications, and it’s easier in Google Scholar to get to the actual publication than it is even through PubMed. (Session 2)  **Weaknesses**  You don’t have time to go to MEDLINE to look up the source literature, at least in most situations; unless you already kind of know what you are looking for. (Session 3)  Another problem with Google Scholar is you get a lot of old stuff and not a lot of new stuff. (Session 2) |
| Printed materials (textbooks, article reprints) | **Strengths**   - Are familiar and consistent; visual memory helps with search and retention - Are respected and credible - Facilitate review of surrounding topics, i.e., no keyhole effect (see MD Consult above) - Are not dependent on new technologies   **Weaknesses**   - Are often less accessible | **Strengths**  That was a consistent source that didn’t change. ‘Cause now if I go to UpToDate, the article has been changed, or … if I go to the literature there is a more recent article and now I’m reading about that, which is good. It’s updating me, but it’s not as hardwired in my mind. … As an intern, I would refer to my MGH pocket guide, and I referred to it throughout my residency. … I knew exactly where to flip to; I didn’t need the index and I knew exactly where on the page to look for that information, and it was sort of just on the tip of my tongue (or my mind) and if I had to look for it I would. But now, when I’m looking at different resources, it doesn’t come back as quickly, that stored memory. (Session 9)  I have some paper textbooks in my office that I use. … I’m really familiar with and like to use. (Session 6)  All that peripheral knowledge that you get from paper text. (Session 9)  If I have time I’ll look it up on either [a] hardcopy textbook I have access to (cause I keep some of those in my office) or I’ll do the online search for one of these things, PubMed sometimes, sometimes UpToDate. (Session 8)  I may go to the Mayo lab book to check that out to be sure of what, what I ordered and what the implication is. (Session 5)  I use the little PDR books. … Because, I don’t have a data phone. (Session 11) |
| Personal notes | **Strengths**   - Are familiar, personalized, and relevant - Are quick - Can be implemented using various technologies   **Weaknesses**   - Are difficult to keep organized, find information - May be less accessible | **Strengths**  I have a file on my Outlook notes that I’ve kept since I was a resident, by topic. And so that’s oftentimes what I use for my kind of peripheral brain. I’ve looked at [it] probably 3 times this morning in clinic just to remind myself what’s the criteria for staging of follicular low-grade lymphoma, or whatever; so I’ve got it all there. I refer to that, and I can refer to it in the room with the patients too, pretty quickly. (Session 9)  It started when I was on a Palm Pilot so I would just take notes on rounds. … I’ve figured out how to get it on my iPad too, so I have it with me at all times, so it’s a matter of taking what I’m used to and making it work with the current technology. (Session 9)  **Weaknesses**  I do have a collection of articles, but how often [do] I reference them? I can’t even keep track of what articles are in there, so I just end up looking it up, elsewhere. (Session 9) |
| Specific Internet sites | **Strengths**   - Offer information useful to patients; empowers patient to answer their own questions (from specific sites) - Offer patient handouts (from specific sites) | **Strengths**  I also sometimes use mayoclinic.com particularly for patient education. And there’s a lot of information there to remind myself of. And showing the patient how to access that information so that they can sort of be empowered to provide their own; where they can go for medical questions that they have. (Session 7)  If I’m looking for something for the patient to take home, I use mayoclinic.com. I type in the diagnosis for them and print it out… Or if I’m having a – go get a something out of the room – “Hey, read this while I’m gone. Let me know if it sounds like you. Do you understand this?” And the treatments are right on there too, so they know I’m doing appropriate things. So I like that for handouts stuff. I use FP Notebook a lot, just because it’s similar to UpToDate, but it’s faster, it’s not so wordy. It’s usually bullet points. (Session 10)  I think you could find almost any procedure you want to show a patient on YouTube. |
| Micromedex | **Strengths**   - Is focused on specific type of information (pharmacotherapy) | **Strengths**  Well, it depends on what it is. I mean, if it’s a drug-related question, you might … go to Micromedex and just look at the doses and drug interactions and side effects and what not. (Session 10)  Micromedex is certainly something I use a lot. Looking for drug interactions (Session 7) |
| Mobile devices | **Strengths**   - Are always available   **Weaknesses**   - Suffer from small screen - Require different applications with change in mobile operating system platform | **Strengths**  The advantage that Epocrates has is it’s in your pocket. So when you’re moving around, I can do it. I can whip it out when I’m in the hallway before I go in the room. … Anywhere I am, I can reach that and then in 2 seconds, I can have an answer. (Session 6)  **Weaknesses**  I don’t frequently use the point of care applications from my portable device but it’s more for, you know, checking email, looking up patients on the list and lab results. … It is kind of hard on the iPhone. (Session 7)  I had a palm pilot with Epocrates, and then … now I have an iPad with Micromedex and Synthesis. (Session 9) |
| Electronic medical record | **Strengths**   - Is integrated into workflow: efficient, relevant   **Weaknesses**   - Is sparsely implemented thus far | **Strengths**  I went to [an online resource]. It took a lot of digging, but I found it. … And then when I went to the Orders [module], it actually popped right up there. (Session 9)  If I know the content is easily available to me, and it’s just a click or two away, I’ll check it very frequently. For instance, I’ve always kind of wished in an ideal world that you get these funny fungal cultures back from bronchoscopy or something like that and the lab report reads out *Candida “blah”*, and I think, “What is that?” If there was just a button that said “Candida blah – AskMayoExpert.” And you could get one click away from a paragraph, “What is this bug”; I mean I’d click it every time. And that’d be the first thing I’d go to. [Other participant: The point of care, you know. Right there.] The point of access of information. (Session 8)  Where you’re looking at the cultures, where you’re ordering the test. If that information is there, you’re much more likely to do it, to use it. (Session 8)  Have it integrated in the workflow—a click away from the scrunchy-look face: “What is that?” (Session 8)  **Weaknesses**  Point of care learning might be useful if our EMR were more functional clinically. There’s not much of anything there that’s useful from that standpoint. If there were … recommendations regarding preventive health, screening, … treatments. … Tying that to the clinical information in front of you (i.e., the patient) might be more helpful. … There is potential for saving time. (Session 4) |
